# Notum as a Crucial Regulator of Matrix Integrity in Dentinogenesis

**DOI:** 10.1002/jcp.70070

**Published:** 2025-07-21

**Authors:** Hwajung Choi, Ju‐Kyung Jeong, Dinuka Adasooriya, Sung‐Won Cho, Eui‐Sic Cho

**Affiliations:** ^1^ Cluster for Craniofacial Development and Regeneration Research, Institute of Oral Bioscience Jeonbuk National University School of Dentistry Jeonju Republic of Korea; ^2^ Division of Anatomy and Developmental Biology, Department of Oral Biology, BK21 FOUR Project Yonsei University College of Dentistry Seoul Republic of Korea

**Keywords:** dentinogenesis, extracellular matrix, Notum, odontoblasts, Wnt/β‐catenin signaling

## Abstract

Dentinogenesis, the formation of dentin, requires precise coordination of cellular differentiation, extracellular matrix synthesis, and signaling regulation. Here, we elucidate the role of Notum, a secreted Wnt inhibitor, in orchestrating these processes during dentin formation. In *Notum* ^*−/−*^ mice, dentin exhibited a thicker yet dysplastic structure with disrupted tubule organization and impaired mineralization, deviating from the functional architecture of healthy dentin. Loss of *Notum* led to excessive activation of Wnt/β‐catenin signaling within the dentin‐pulp complex and enhanced expression of odontogenic genes, including *dentin sialophosphoprotein* (*Dspp*), and *dentin matrix protein 1* (*Dmp1*). However, this upregulation was uncoupled from proper extracellular matrix composition and mineralization, indicating that initial odontoblast differentiation alone is insufficient for functional dentin formation. At the molecular level, *Notum* deficiency disrupted matrix integrity, characterized by reduced collagen organization and increased expression of non‐collagenous matrix proteins such as bone sialoprotein (Bsp). Collectively, these findings highlight Notum as a critical modulator that fine‐tunes Wnt/β‐catenin signaling to coordinate cellular differentiation with matrix organization during dentinogenesis. Therapeutic targeting Notum may offer new strategies for restoring dentin integrity and enhancing regenerative outcomes.

## Graphical abstract

With Notum deficiency, mouse dentin exhibits a thicker yet dysplastic structure with disrupted tubular organization, resulting in functionally compromised dentin. Notum precisely regulates Wnt/β‐catenin signaling within the dentin‐pulp complex to support dentin formation. In conclusion, Notum plays a critical role in maintaining structural integrity during dentinogenesis by orchestrating extracellular matrix composition and modulating key signaling pathways.

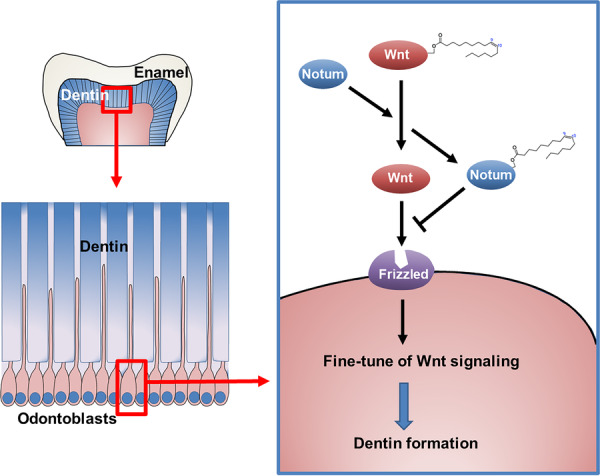

## 1 Introduction

As the second hardest tissue of the human body, dentin provides structural support for enamel, acting as a resilient shock‐absorber against mechanical stress (Goldberg 2011; Smith et al. 2012). Mature dentin boasts a complex hierarchical organization, with dentinal tubules, as a key feature. These tubules extend radially from the pulp to the dentin‐enamel junction, housing the odontoblastic processes (Butler and Ritchie 1995; Weerakoon et al. 2022). Dentinal tubules functionally contribute to detect mechanical and thermal changes, making them integral to both the physiological and sensory aspects of tooth function (Butler and Ritchie 1995; Goldberg 2011; Shinno et al. 2016). Dentinogenesis is primarily driven by odontoblasts, which reside at the periphery of the dental pulp and migrate centripetally, incrementally depositing the dentin matrix to form the dentin structure (Couve et al. 2013; Jing et al. 2022; Jussila and Thesleff 2012). Unlike enamel, dentin remains dynamic throughout life due to its ability to regenerate and repair in response to stimuli such as wear, trauma, and decay (Smith et al. 2012).

The dentin matrix is primarily composed of collagenous and non‐collagenous proteins, which form a scaffold for mineral deposition (Butler and Ritchie 1995; Smith et al. 2012). Type I collagen (Col I) constitutes approximately 90% of the organic matrix, providing tensile strength and a framework for the nucleation of hydroxyapatite crystals. The remaining 10% of the organic matrix is composed of non‐collagenous proteins, including dentin sialophosphoprotein (Dspp), and dentin matrix protein 1 (Dmp1). Proper matrix composition and structural organization of these components are closely associated with the mechanical properties of dentin (Bertassoni 2017; Kinney et al. 2003; Weerakoon et al. 2022). The spatiotemporal regulation of canonical Wnt signaling is critical for coordinating the proliferation and differentiation of dental epithelial and mesenchymal cells required for proper tooth development (Bae et al. 2015; Yamashiro et al. 2007). Disruption of canonical Wnt signaling at early developmental stages results in arrested odontogenesis (Chen et al. 2009). Aberrant β‐catenin expression in odontoblasts leads to malformation of the dentin structure (Kim et al. 2011). Although modulation of Wnt signaling has been explored as a strategy for dentin regeneration (Aurrekoetxea et al. 2016; Hunter et al. 2015), restoring dentin with appropriate structural and functional properties remains a substantial challenge. Notum, a secreted palmitoleoyl‐protein carboxylesterase, exerts its effects by depalmitoylating Wnt proteins, rendering them inactive and thereby inhibiting Wnt signaling (Kakugawa et al. 2015). Notum is primarily expressed in developing mouse teeth, specifically within the odontoblast progenitors and preodontoblasts near the crown and root regions, including enamel knots and dental papilla (Adasooriya et al. 2024; Krivanek et al. 2020; Yang et al. 2021). *Notum* deficiency leads to dysplastic dentin and short root formation in mice, with phenotypic abnormalities extending beyond the dentition to other organs, including the kidneys and bones (Brommage et al. 2019; Vogel et al. 2016). Although the previous study highlights its essential role in dentinogenesis, the precise regulatory functions of Notum remain undefined.

In this study, we investigated the role of Notum in dentinogenesis, which underscores the intricate interplay between cellular signaling, matrix synthesis, and tissue structure. Although *Notum* is transiently expressed during the early stages of odontoblast differentiation, it plays a sustained functional role in preserving odontoblast identity and regulating proper matrix composition and structural organization throughout dentinogenesis. As research continues to elucidate the molecular mechanisms underlying dentin formation, targeting Notum may advance dental health and regenerative therapies.

## 2 Materials and Methods

### 2.1 Generation of *Notum‐*Knockout Mice

All procedures were performed in accordance with the U.S. National Institutes of Health Guidelines on the Use of Laboratory Animals. All experimental procedures were approved by the Animal Welfare Committee of Jeonbuk National University. *Notum*‐knockout mice (*Notum* ^*em1(IMPC)Tcp*^) were purchased from the international mouse phenotyping consortium (www.mousephenotype.org) and mated with C57BL/6 N wild‐type mice to obtain F1 mice. F2 and above generations of mice (*Notum* ^*+/+*^ and *Notum* ^*−/−*^) were used for the study. Genotypes of the mouse offspring were determined as previously described (Adasooriya et al. 2024).

### 2.2 Tissue Preparation, Histology, and Histomorphometry

Mice were killed and their mandibles carefully dissected for histological analysis. Tissues were fixed in 4% paraformaldehyde (Sigma‐Aldrich, St Louis, MO, USA) and decalcified in 10% ethylenediaminetetraacetic acid for 2–4 weeks at 4°C. The decalcified tissues were dehydrated through a graded ethanol series, embedded in paraffin, and sectioned at a 5‐μm thickness using standard histological procedures as formerly described (Kim et al. 2013). Slides were stained with hematoxylin and eosin (H&E) and trichrome staining solution (Sigma‐Aldrich). Crown dentinal area was measured in the H&E‐stained frontal sections of mandibular first molars using ANALYSIS software (Soft Imaging System GmbH, Muenster, Germany). The measurements were performed with three representative slides from each group.

### 2.3 Immunohistochemistry and In Situ Hybridization

For immunohistochemistry, tissue sections were treated with 3% hydrogen peroxide and incubated with rabbit polyclonal antibodies against β‐catenin (Thermo Fisher Scientific, Waltham, MA, USA), an activated form of β‐catenin lacking phosphorylation at S33/37/Thr41 (Cell Signaling Technologies, Danvers, MA, USA), Dsp (Santa Cruz Biotechnology, Dallas, TX, USA), Dmp1 (Takara Bio, Shiga, Japan), Col I (Abcam, Cambridge, UK), and bone sialoprotein (Bsp) (Takara Bio). The rabbit‐specific HRP/DAB‐detection immunohistochemistry kit (Abcam) was used according to the manufacturer's instructions. In situ hybridization was performed with the RNAscope® 2.5 High‐Definition Assay‐Brown (Advanced Cell Diagnostics, Newark, CA, USA) according to the user manual 322452 (FFPE sample preparation and pretreatment) and 322310 (RNAscope® 2.5 High‐Definition Detection Reagent‐Brown user manual) provided by the manufacturer. The following RNA probes were used: Mm‐*Notum* (1814611, targeting NM_175263.4, nucleotide 381‐689), Mm‐*Dickkopf WNT signaling pathway inhibitor 1* (*Dkk1*, 402521, targeting NM_010051.3, nucleotide 294‐1334), Mm‐*sphingomyelin phosphodiesterase 3* (*Smpd3*, 815591, targeting NM_021491.3, nucleotide 1348‐2252), Mm‐*Dspp* (448301, targeting NM_010080.2, nucleotide 355‐1420), Mm‐*Dmp1* (441171, targeting NM_016779.2, nucleotide 689‐1543), and Mm‐*Axin2* (400331, targeting NM_015732.4, nucleotide 330‐1287). Images were taken using an Olympus BX43 microscope equipped with an Olympus DP23 digital camera (Olympus Corp., Tokyo, Japan).

### 2.4 Scanning Electron Microscopy (Sem) Analysis

For SEM analysis, the mandibles were dissected at postnatal week 13 and fixed in 70% ethanol at room temperature for 24 h. The tissue specimens were dehydrated through a graded ethanol series, embedded in methylmethacrylate without decalcification. To avoid the risk of estimation error due to the curved shape of mouse molar tubules, tissue specimens were prepared from the region immediately above the pulpal horn by grinding with three different surface preparations: 600‐, 180‐, and 120‐grit SiC paper (Buehler, Lake Bluff, IL, USA). The surfaces were acid‐etched with 10% formic acid for 10 s, followed by 5% sodium hypochlorite for 20 min. The specimen surfaces were sputter‐coated with platinum after drying and examined with a scanning electron microscope (SU3900; Hitachi, Tokyo, Japan) under 5.0‐kV conditions.

### 2.5 Cell Culture, Transfection, and Stable Cell Lines with shRNA

MDPC‐23 cells (Hanks et al. 1998), a murine dental papilla cell line, were maintained in growth media consisting of Dulbecco's modified Eagle medium (Invitrogen, Carlsbad, CA, USA) supplemented with 10% fetal bovine serum (Invitrogen), and 100 IU/mL of penicillin‐100 μg/mL of streptomycin (Invitrogen). To generate retroviral particles, shRNA against mouse *Notum* (TR512881) or control shRNA (TR30013) was purchased from OriGene Technologies (Rockville, MD, USA). The establishment of stable cell lines by viral transductions was performed as previously described (Choi et al. 2017).

### 2.6 Statistical Analysis and Supporting Information

Data are presented using mean ± standard error of the mean values of three or more separate experiments. Normal data with equal variance were analyzed using Student's *t*‐test, and *p* < 0.05 was considered statistically significant. Detailed descriptions for other experimental materials and methods are provided in the Supporting Methods.

## 3 Results

### 3.1 Expression Pattern of *Notum* in Developing Mouse Teeth and Its Deletion in *Notum* ^*−/−*^ Mice

**Figure 1:**
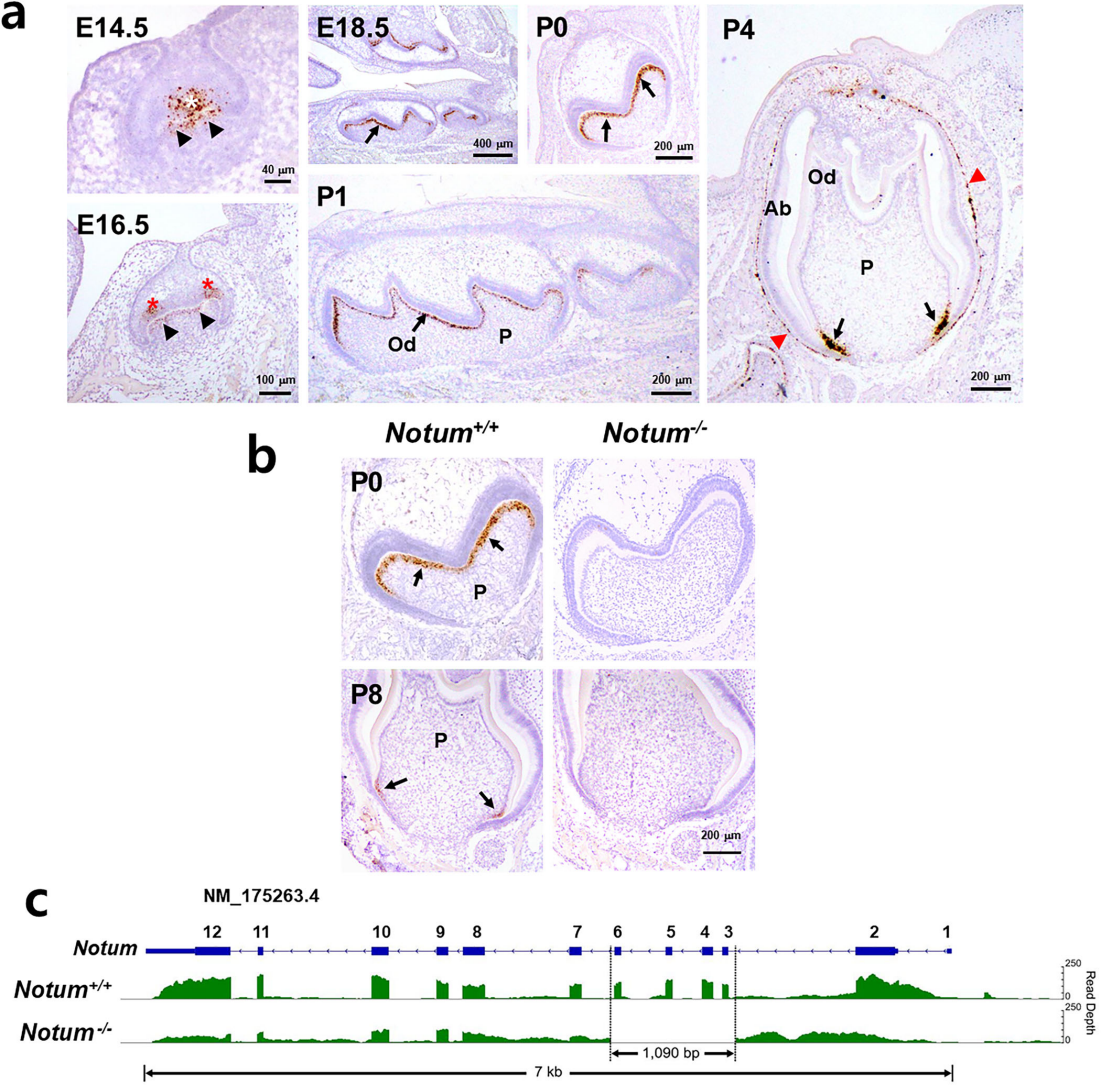
Expression pattern of *Notum* in developing mouse teeth and its deletion in *Notum* ^*−/−*^ mice. (a) In situ hybridization images of *Notum* genes in the frontal sections of mandibular molars at embryonic day (E) 14.5, E16.5, E18.5, postnatal day 0 (P0), P1, and P4. *Notum* is expressed in the primary enamel knot (EK) at E14.5 (white asterisk), secondary EK at E16.5 (red asterisks), and in the thin outer layer of the dental papilla (black arrowheads). Black arrows and red arrow heads highlight *Notum* expression in the odontoblasts and outer dental epithelial cells of mouse molars, respectively. (b) In situ hybridization images showing *Notum* expression in the frontal sections of mandibular molars at P0 and P8. Black arrows indicate that *Notum* expression is restricted in preodontoblasts and differentiating odontoblasts near the apex in *Notum* ^*+/+*^ mice, which is disappeared in *Notum* ^*−/−*^ mice. (c) A map illustrating the reduced read depth and deletion of exons 3 to 6 in the *Notum* region of *Notum* ^*−/−*^ mice compared to *Notum* ^*+/+*^ mice, as analyzed through RNA sequencing. Ab, ameloblasts; Od, odontoblasts; P, pulp.

To investigate the role of *Notum* in dentin formation, we examined its expression during tooth development. During the early embryonic stage, *Notum* was predominantly expressed in the enamel knot, whereas postnatally, its expression shifted to preodontoblasts in the mesenchyme and later localized primarily in differentiating odontoblasts (Figure 1a). *Notum* expression was no longer observed in fully differentiated odontoblasts at the coronal region at P4. At postnatal day 0 (P0), strong *Notum* expression was detected in odontoblasts within the coronal region of *Notum* ^*+/+*^ mice but was absent in *Notum* ^*−/−*^ mice. Its loss of expression was evident in differentiating odontoblasts near the apex in *Notum* ^*−/−*^ mice at P8 (Figure 1b). These results indicate that *Notum* expression is transient, restricted to preodontoblasts and early differentiating odontoblasts, and is not maintained for full differentiation of odontoblasts. RNA‐sequencing analysis confirmed the effective knockout of *Notum* in *Notum* ^*−/−*^ mice, showing a marked reduction in read depth and deletion of exons 3 to 6 (Figure 1c).

### 3.2 Dysplastic Dentin Formation With Disrupted Tubular Structure in *Notum* ^*−/−*^ Mice

**Figure 2:**
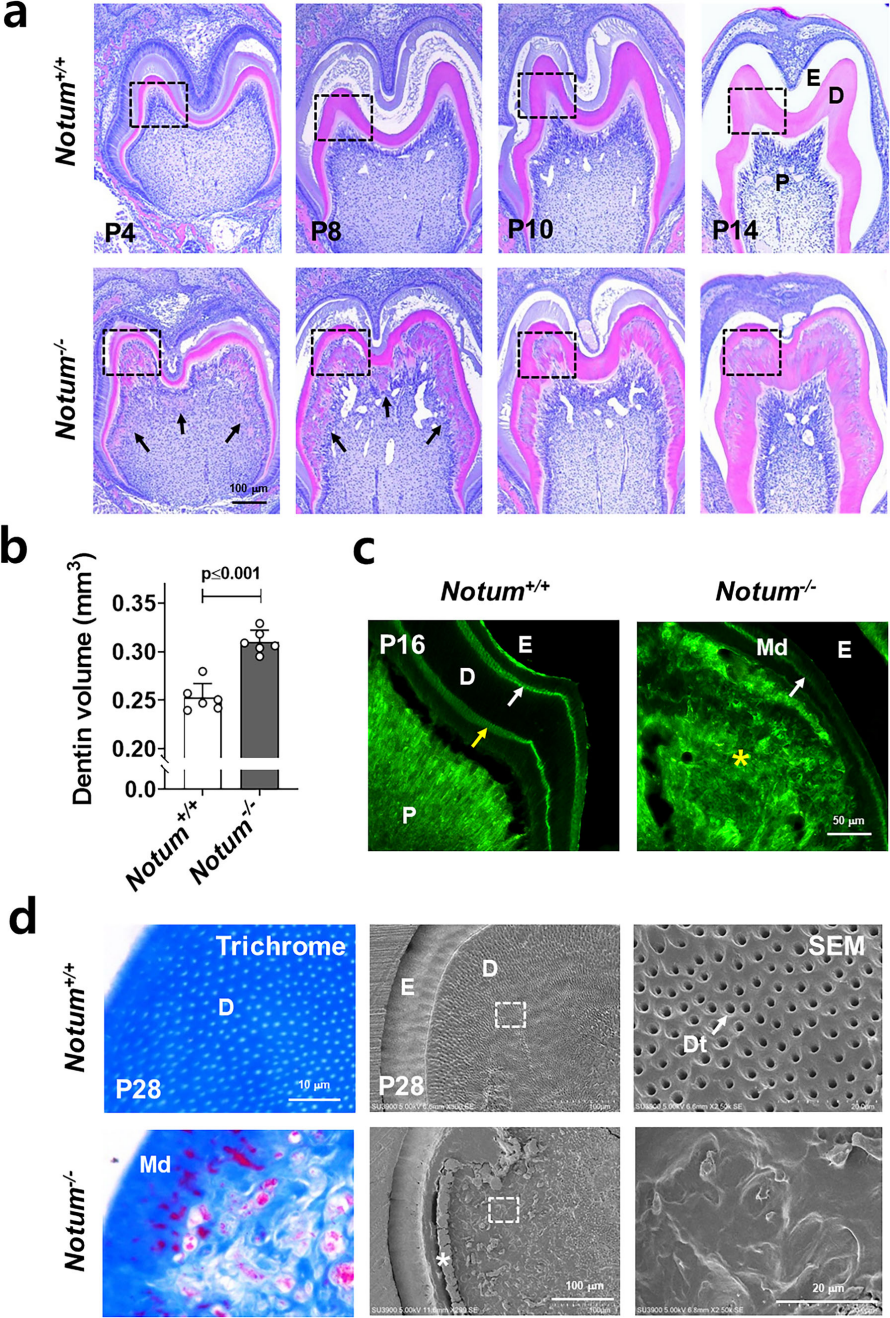
Dysplastic dentin formation with disrupted tubular structure in *Notum* ^*−/−*^ mice. (a) Frontal sections of mouse tooth tissue from P4 to P14 stained with H&E, showing differences in dentin structure between *Notum* ^*−/−*^ and *Notum* ^*+/+*^ mice. Black arrows indicate ectopic extracellular matrix deposition by odontoblasts that have lost polarity in *Notum* ^*−/−*^ mice. Magnified views of the black‐dot boxed area were present in Figure S1a. (b) Quantification of dentin volume using micro‐CT (*n* = 6). Statistical significance is indicated by *p*‐values. (c) Sagittal tissue sections of molars from calcein‐injected *Notum* ^*−/−*^ and *Notum* ^*+/+*^ mice at P16. White arrows indicate the first calcein labeling (P7), while the yellow arrow marks the second labeling (P14). The yellow asterisk denotes the absence of clear demarcation in the second calcein labeling of *Notum* ^*−/−*^ molars, coinciding with the formation of osteodentin. (d) Horizontal sections of 28‐day‐old mouse molar stained with trichrome and observed by SEM. The white asterisk (*) indicates areas of resin infiltration between mantle dentin and circumpulpal dentin in the molar of *Notum* ^*−/−*^ mice during tissue processing for SEM. The far‐right images show a magnified view of the white‐boxed area in the central SEM images. P, pulp; D, dentin; E, enamel; Ab, ameloblasts; Od, odontoblasts; Md, mantle dentin; Dt, dentinal tubule.

The absence of the *Notum* gene in *Notum* ^*−/−*^ mice resulted in marked alterations in dentin formation, mainly characterized by changes in the physical structure. Figure 2a presents the temporal sequence of dentin formation across various stages of postnatal development in both *Notum* ^*+/+*^ and *Notum* ^*−/−*^ mice. In *Notum* ^*+/+*^ mice, odontoblasts established tubular structures as dentin developed in an organized pattern (Figure S1a). In contrast, in *Notum* ^*−/−*^ mice, although mantle dentin initially formed correctly, the polarity of odontoblasts was subsequently disrupted, leading to excessive secretion of extracellular matrix toward the pulp cavity. As a result, odontoblasts became entrapped within the matrix, forming a bone‐like tissue (Figure S1a). The deposited matrix progressively accumulated and eventually merged with the pre‐existing mantle dentin (Figures 2a, S1a and b). Interestingly, in the dentin of *Notum* ^*−/−*^ mice, toward the end of crown dentin formation, odontoblasts appeared to realign between the dentin and the pulp while maintaining their cellular polarity (Figure 2a). The overall dentin layer in *Notum* ^*−/−*^ mice was thicker than in *Notum* ^*+/+*^ mice, yet its structural integrity was severely compromised due to tubule and matrix disorganization (Figures 2a, S1a and c). Quantitative analysis confirmed a significant increase in dentin volume in *Notum* ^*−/−*^ mice, suggesting enhanced dentin matrix deposition upon *Notum* deficiency (Figure 2b). To experimentally assess dentin thickening, calcein dye labeling was also performed. In *Notum* ^*+/+*^ mice, sequential calcein injections produced two distinct fluorescent lines, indicating incremental deposition. In contrast, *Notum* ^*−/−*^ mice exhibited a faint line of first calcein labeling, while the second labeling was diffusely distributed and intermingled with cells within bone‐like tissue (Figure 2c). Consistent with molar defects in *Notum* ^*−/−*^ mice, incisors also exhibited dysplastic dentin formation, indicating that *Notum* deficiency disrupts dentin formation across multiple tooth types (Figure S1d). We also performed a comparative analysis of dentin structure using trichrome staining and SEM prepared by horizontal sectioning of mouse teeth (Figure 2d). In *Notum* ^*+/+*^ mice, dentinal tubules were typically aligned in a regular pattern. In contrast, *Notum* ^*−/−*^ mice exhibited a marked reduction in tubule number and irregularly shaped tubule formation. Although the dentin in *Notum* ^*−/−*^ mice was thicker, its quality was definitely inferior to that of *Notum* ^*+/+*^ mice.

### 3.3 Dysregulated Gene Expression in the Teeth of *Notum* ^*−/−*^ Mice

**Figure 3:**
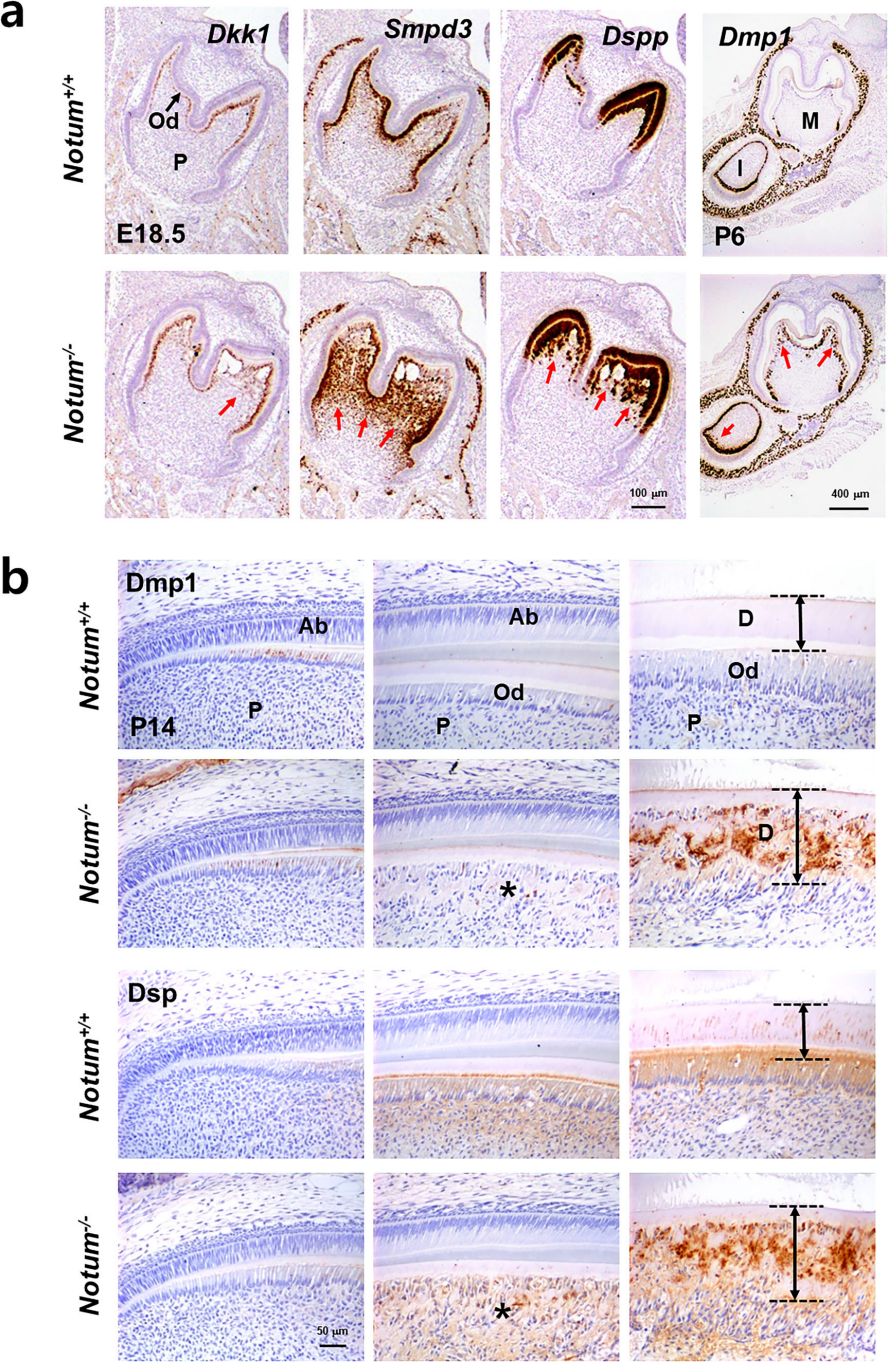
Dysregulated gene expression in the teeth of *Notum* ^*−/−*^ mice. (a) In situ hybridization images depicting RNA expression levels of specific genes in E18.5 and P6 dental tissues acquired by frontal sectioning. Red arrows highlight upregulated gene expression in the teeth of *Notum* ^*−/−*^ mice. (b) Immunohistochemical analysis of sagittal sections from mouse incisor tissues at P14 with specific antibodies to compare protein localization in *Notum* ^*‐/‐*^ and *Notum* ^*+/+*^ mice. Each incisor panel represents distinct stages of odontogenesis, from early differentiation to initial secretion and active matrix deposition phases, respectively. Asterisks indicate ectopic extracellular matrix deposition by odontoblasts that have lost polarity in *Notum* ^*−/−*^ mice, contributing to dentin dysplasia. Double‐headed arrows denote dentin thickness. Od, odontoblasts; P, pulp; M, molar; I, incisor; Ab, ameloblasts; D, dentin.

As demonstrated by in situ hybridization in Figure 3a, odontoblast‐specific genes, including *Dkk1*, *Smpd3*, *Dspp*, and *Dmp1* were analyzed using tissue sections from developing teeth (Figure 3a). As observed in the early tooth germ at embryonic day 18.5 (E18.5), *Notum* ^*−/−*^ mice exhibited broader cusp tips in the molars compared to the sharp cusps in *Notum* ^*+/+*^ mice. In *Notum* ^*−/−*^ mice, the RNA expression of these genes was markedly elevated and widely distributed within the coronal region of the dentin‐pulp complex compared to that in *Notum* ^*+/+*^ mice. Considering that *Notum* expression is typically restricted to preodontoblasts and odontoblasts, this upregulation and broad distribution of odontogenic genes suggest that the cellular properties of odontoblasts were dramatically altered by *Notum* deficiency (Komori 2010; Miyazaki et al. 2008). Consistent with these results, immunohistochemical staining results with the dental tissues from mouse incisors also exhibited an elevated protein expression of Dmp1 and Dsp in abnormal dentin matrix formed by *Notum* deficiency (Figure 3b).

### 3.4 Activation of β‐catenin and Impaired Dentin Matrix Composition in the Teeth of *Notum* ^*−/−*^ Mice

**Figure 4:**
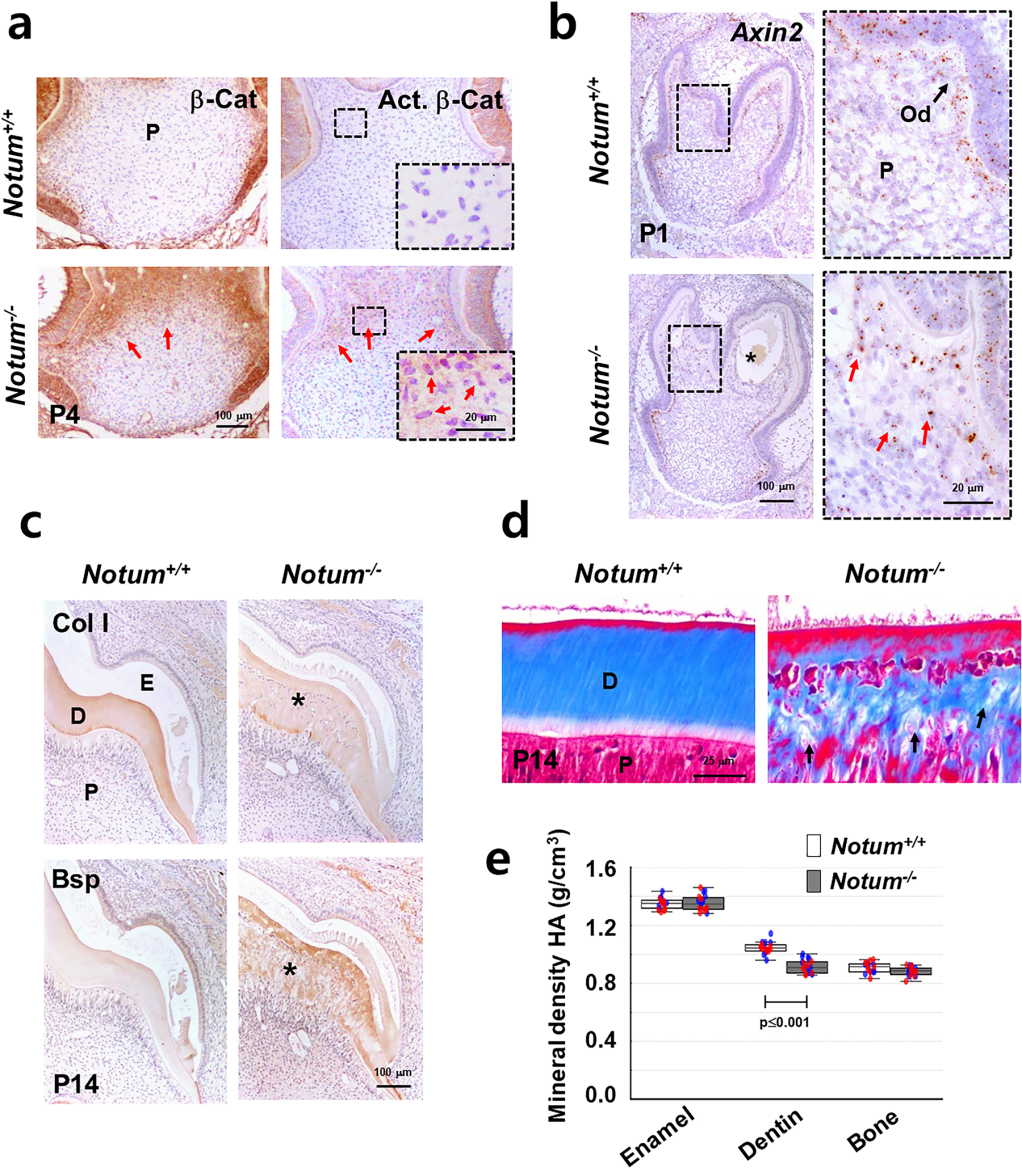
Activation of β‐catenin and impaired dentin matrix composition in the teeth of *Notum* ^*−/−*^ mice. (a) Immunohistochemical analysis of protein localization in mouse dental tissues at P4 acquired by frontal section. Red arrows indicate increased protein expression in the teeth of *Notum* ^*−/−*^ mice. (b) In situ hybridization images depicting expression levels of *Axin2* genes in mouse dental tissues at P1 acquired by frontal section. Red arrows highlight upregulated gene expression in the teeth of *Notum* ^*−/−*^ mice. The right images present an enlarged view of the black‐boxed area within the left images. (c) Immunohistochemical comparison of protein‐expression patterns in the sagittal sections of P14 mouse second molars. Asterisks indicate altered staining patterns of proteins in the dentin of *Notum* ^*−/−*^ tissues. (d) Trichrome staining of sagittal sections of P14 mouse incisors, highlighting structural differences in dentin matrix. Black arrows indicate sparser collagen architecture in the dentin of *Notum* ^*−/−*^ mice. (e) The mineral density of enamel, bone, and dentin was measured using micro‐CT images of mandibular tissues from P35 mice. Blue and red dots represent the measured values from the left and right hemimandibles in each group, respectively. Significance was assigned for *p*‐values as indicated. E, enamel; D, dentin; P, pulp; Od, odontoblasts.

The immunohistochemical staining results revealed that *Notum* deficiency leads to a sustained Wnt/β‐catenin signaling within the coronal region of the dentin‐pulp complex (Figure 4a and b). Stronger signal intensity of total β‐catenin and its activated form, lacking phosphorylation at S33/37/Thr41 and consequently avoiding degradation through Gsk3β (Behrens et al. 1998), was also observed in the coronal region of the dentin‐pulp complex of *Notum* ^*−/−*^ mice compared to in *Notum* ^*+/+*^ mice (Figure 4a). Consequently, the absence of *Notum* led to enhanced expression of *Axin2*, a known target of Wnt/β‐catenin signaling (Jho et al. 2002) (Figure 4b). In a mouse model where β‐catenin was specifically activated in odontoblasts, as previously observed, the expression of extracellular matrix proteins was notably enhanced (Kim et al. 2011). Immunohistochemical analysis also revealed a reduction in Col I expression in dentin of *Notum* ^*−/−*^ mice, whereas Bsp expression was markedly increased (Figure 4c). Trichrome staining further highlighted disorganized and sparser collagen architecture in *Notum* ^*−/−*^ compared to *Notum* ^*+/+*^ mice (Figure 4d). Furthermore, micro‐CT analysis revealed a significant reduction in dentin mineral density in *Notum* ^*−/−*^ mice, whereas enamel and bone remained unaffected (Figure 4e).

### 3.5 Impaired Matrix Protein Expression and Mineralization in *Notum*‐Knockdown Odontogenic Cells

**Figure 5:**
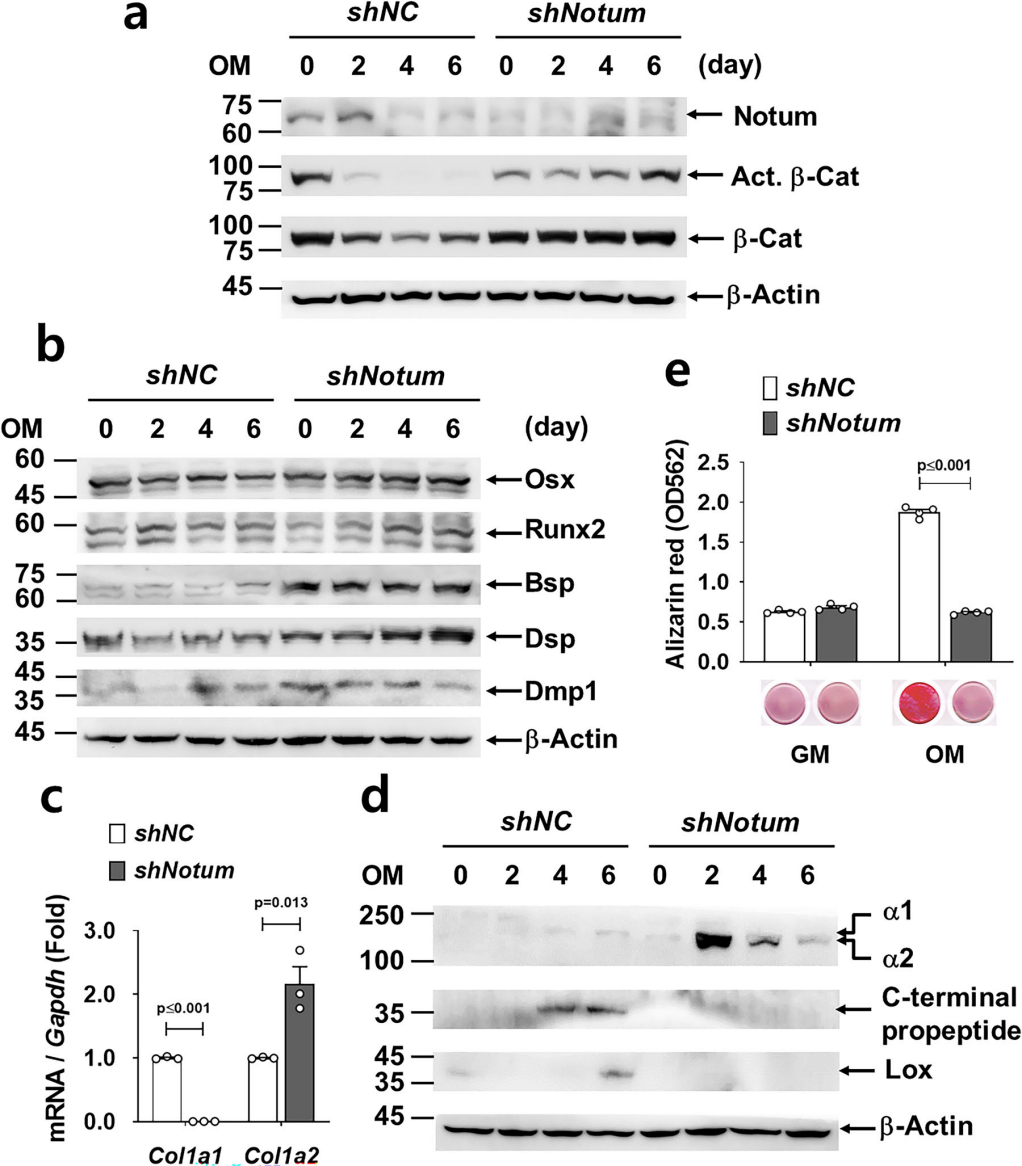
Impaired matrix protein expression and mineralization in *Notum*‐knockdown odontogenic cells. (a) Western blot analysis comparing protein levels during differentiation between *shNotum* and *shNC* cells. The samples were derived from the same experiment and gels/blots were processed under the same experimental conditions. β‐actin was used as a loading control. The original full‐size blots are presented in Figure S4. (b) Western blot analysis depicting temporal protein‐expression profiles during differentiation in *shNotum* and *shNC* cells. The original full‐size blots are presented in Figure S5. (c) Quantitative RT‐PCR analysis comparing RNA‐expression levels of *Col I* subunits in *shNotum* and *shNC* cells. (d) Western blot analysis showing temporal expression changes of collagen fibrillogenesis‐related proteins during differentiation in *shNotum* and *shNC* cells. The samples were derived from the same experiment and gels/blots were processed under the same experimental conditions. β‐actin was used as a loading control. The original full‐size blots are presented in Figure S6. (e) Alizarin Red staining and subsequent quantification were performed to assess extracellular matrix mineralization in *shNotum* and *shNC* cells. β‐Cat, β‐catenin; Act. β‐Cat, activated form of β‐catenin; α1, procollagen I α1; α2, procollagen I α2; Lox, Lysyl oxidase; OM, osteogenic media; GM, growth media. Statistical significance is indicated by *p*‐values as shown in the graph.

We established *Notum*‐knockdown odontogenic cell lines using MDPC‐23 cells with stable *shRNA* transduction. Western blot analysis revealed the expression of Notum during the early stages of differentiation in the control *shNC* cells, consistent with previous findings (Zhao et al. 2024). In contrast, *shNotum* cells exhibited a marked reduction in Notum protein levels, effectively suppressed by *shRNA* targeting the *Notum* gene at these stages (Figure 5a). In the control *shNC* cells, protein levels of activated β‐catenin were initially high but gradually declined during differentiation. In contrast, in *shNotum* cells, activated β‐catenin levels were initially lower than those in *shNC* cells but progressively increased. These results suggest an initial compensatory effect by other Wnt inhibitors such as Dkk1 (Figure S2), followed by a delayed but prolonged inhibitory role of Notum in Wnt/β‐catenin signaling. When cells were differentiated in osteogenic medium (OM), the protein expression of osterix (Osx) and runt‐related transcription factor 2 (Runx2) showed a slight decrease during differentiation in control cells. However, in *shNotum* cells, their protein levels exhibited a modest increase. Additionally, the expression levels of Bsp, Dsp, and Dmp1 were generally elevated in *shNotum* cells, suggesting an altered regulatory mechanism of differentiation in the loss of *Notum* (Figure 5b). The protein expression results were generally consistent with the RNA expression pattern. However, the alteration in gene expression induced by *Notum* deficiency were not restored by supplementation with soluble Notum under our in vitro experimental conditions (Figure S3). To better understand the defects in the dentin matrix caused by *Notum* deficiency, we examined alterations in collagen synthesis at the molecular level using odontogenic cells. qRT‐PCR analysis demonstrated a differential regulation of *collagen* genes; downregulation of *Col1a1* and upregulation of *Col1a2* in *shNotum* cells during differentiation (Figure 5c). Consistent with these results, as observed by Western blot analysis, procollagen α1 and α2 were regulated similarly in *shNotum* cells (Figure 5d). The absence of C‐terminal propeptide cleavage and lysyl oxidase (Lox) expression in *shNotum* cells indicated defective procollagen processing and impaired fibrillogenesis, disrupting collagen matrix assembly and stabilization compared to *shNC* cells. Collagen assembly directly influences the mineralization of the matrix (Añazco et al. 2023). *Notum* deficiency in odontogenic cells resulted in a significant reduction in their mineralization capacity, as demonstrated by Alizarin Red staining (Figure 5e).

## 4 Discussion

Functional dentin formation involves the establishment of dentinal tubules, which facilitate fluid movement and nerve signaling essential for tooth sensitivity and function (Goldberg 2011). The presence, orientation, and distribution of these tubules create a complex microstructure that critically influences the mechanical properties of dentin (Han et al. 2012; Weerakoon et al. 2022). In *Notum* ^*−/−*^ mice, dentin exhibits a thicker yet dysplastic structure with disrupted tubule organization, deviating from the well‐defined tubular network of healthy dentin. At the molecular level, the disrupted balance between collagen subunits, along with the reduced expression of Lox, suggests impaired collagen fibrillogenesis and structural assembly within the dentin matrix. Concurrently, the elevated expression of Bsp indicates a shift in the dentin matrix toward bone‐like characteristics. Although the regulatory role of *Notum* in dentin formation has been suggested, its precise expression pattern and detailed function have remained poorly understood. In this study, we found that *Notum* is expressed during the early stages of odontoblast differentiation and, although it does not affect mantle dentin formation, it is essential for regulating overall processes of circumpulpal dentin formation, including matrix secretion and structural organization. These results indicate that Notum primarily attenuates excessive Wnt/β‐catenin signaling, thereby facilitating incremental and centripetal deposition of the dentin matrix, which ensures proper tubule formation and subsequent mineralization.

The correlation between thickened dentin formation and increased Wnt/β‐catenin signaling was previously demonstrated by conditional transgenic mice (*OC‐Cre; Catnb* ^*lox(ex3)/+*^) with stabilized β‐catenin in the dental mesenchyme (Kim et al. 2011). Enforced activation of β‐catenin in odontoblasts led to excessive dentin formation. However, the excessive dentin was formed by prematurely differentiated odontoblasts, resulting in globular dentin with hypomineralization. Our study highlights the importance of tightly regulated Wnt/β‐catenin signaling in functional dentin formation. While Wnt signaling is critical for odontogenic differentiation of neural crest‐derived mesenchymal cells during tooth development (Jing et al. 2022), odontogenic differentiation by this signaling does not guarantee the formation of functional dentin, as is evident in cases where mineralization is suboptimal and the characteristic tubular structures are absent. Our findings indicate that the fine‐tuning of Wnt/β‐catenin signaling by Notum is required not only for odontoblast differentiation but also for the coordinated synthesis and organization of extracellular matrix components necessary for functional dentin.

Interestingly, although *Notum* is transiently expressed during the early stages of odontoblast differentiation, its influence extends into later phases by regulating matrix composition and architecture, thereby contributing to the establishment of the unique characteristics of dentin. In *Notum* ^*−/−*^ mice, initial odontoblast differentiation appears intact; however, during later stages, these cells exhibit a phenotypic shift toward osteoblast‐like state. This observation suggests that Notum plays a key role dentinogenesis by attenuating Wnt/β‐catenin signaling, thereby maintaining odontoblast identity and ensuring the formation of structurally organized dentin. Similarly, Runx2 is predominantly expressed in preodontoblasts and immature odontoblasts, and its overexpression in transgenic mice leads to the loss of typical odontoblastic morphology and the formation of bone‐like dentin (Miyazaki et al. 2008). Runx2 has been implicated in the inhibition of terminal differentiation of odontoblasts and in promoting their transdifferentiation into osteoblast‐like cells (Miyazaki et al. 2008). Collectively, these findings underscore the importance of early transient regulatory signals in directing the long‐term functionally organized formation of dentin.

Notum exhibits several unique regulatory features in cellular signaling. A previous study reported that Notum exerts bidirectional effects (Zhao et al. 2024), probably modulating cellular processes in a context‐dependent manner. These findings indicate that precise temporal and dosage regulation of Notum is critical during dentin formation. Furthermore, despite being a secreted protein, supplementation with soluble Notum failed to rescue the altered gene expression in *Notum*‐knockdown odontogenic cells, suggesting that its full biological activity may depend on specific cellular contexts or microenvironmental cues. These observations imply the complex regulatory role of Notum in coordinating signaling dynamics during dentin formation.

Collectively, our studies elucidate the multifaceted role of Notum in orchestrating both cellular differentiation, and matrix composition to regulate dentin formation. Our findings highlight how *Notum* deficiency leads to a cascade of pathological changes, culminating in defective dentin that lacks functional integrity. Therapeutic targeting of Notum to modulate Wnt/β‐catenin signaling and reinforce matrix integrity presents a promising strategy for advancing dentin repair and regeneration.

## Author Contributions

Hwajung Choi, Sung‐Won Cho, and Eui‐Sic Cho conceived and designed the research. Ju‐Kyung Jeong and Dinuka Adasooriya performed the animal experiments. The histological tissue analysis and scoring were conducted by Hwajung Choi and Ju‐Kyung Jeong The statistical analysis and interpretation of the results were performed by Hwajung Choi, Sung‐Won Cho, and Eui‐Sic Cho The manuscript was principally written and revised by Hwajung Choi, Sung‐Won Cho, and Eui‐Sic Cho All the authors critically reviewed the manuscript for important intellectual content and approved the final submitted manuscript.

## Conflicts of Interest

The authors declare no conflicts of interest.

## Supporting information

JCP‐24‐2010‐ Supporting Information.

## Data Availability

The data sets used and/or analyzed during the current study are available from the corresponding author on reasonable request.
