# Supplementary material for: Notum as a Crucial Regulator of Matrix Integrity in Dentinogenesis
Source: J Cell Physiol. 2025 Jul 21;240(7):e70070. doi: 10.1002/jcp.70070 (PMC12277939; doi:10.1002/jcp.70070)

Supporting Information

Notum as a Crucial Regulator of Matrix Integrity in Dentinogenesis

Hwajung Choi[1], Ju-Kyung Jeong[1], Dinuka Adasooriya[2], Sung-Won Cho[2,*], Eui-Sic Cho[1,*]

[1]Cluster for Craniofacial Development and Regeneration Research, Institute of Oral Bioscience, Jeonbuk National University School of Dentistry, Jeonju, Republic of Korea

[2]Division of Anatomy and Developmental Biology, Department of Oral Biology, BK21 FOUR Project, Yonsei University College of Dentistry, Seoul, Republic of Korea

Running title: Notum regulates dentinogenesis

[*]Author for correspondence
Sung-Won Cho, DDS, PhD
Division of Anatomy and Developmental Biology
Yonsei University College of Dentistry
E-mail: chosome1@yuhs.ac

Eui-Sic Cho, DDS, PhD
Cluster for Craniofacial Development and Regeneration Research
Jeonbuk National University School of Dentistry
E-mail: oasis@jbnu.ac.kr

Supporting Methods

Micro-computed tomography and mineral density measurement

Mandibles of *Notum*$^{+/+}$ and *Notum*$^{-/-}$ P35 mice were dissected and fixed in 4% Paraformaldehyde (PFA) in PBS. Micro-computed tomography (micro-CT) images were obtained using a micro-CT scanner (Skyscan1173, Bruker, Belgium) at 130 kV and 60 µA with the 0.25 g/cm^3 and 0.75 g/cm^3 Phantom rods. The data were reconstructed using Nrecon (Version 1.6) with consistent parameters. CT-Analyser (CTAn) (Version 1.17.7.1, SkyScan, Bruker, Belgium) was calibrated with the phantom rods, and the enamel, dentin, and bone mineral density were measured (n = 6 hemimandibles per group). The enamel and dentin mineral density were measured in three different points covering 10 slices at the mesial, distal, and lingual regions of the mandibular first molar. The bone mineral density was measured at 3 points covering 10 slices at the mandibular diastema region.

Generation of *Notum* deletion map

The deletion map was created using the Binary Alignment Map (BAM) files obtained from RNA sequencing data from our previous study involving *Notum* knockout mice (Adasooriya et al., 2024), where the raw data were publicly available at the National Center for Biotechnology Information (NCBI) Gene Expression Omnibus (GEO) under accession number GSE255946. BAM files corresponding to the RNA sequences E16.5 *Notum*$^{+/+}$ and *Notum*$^{-/-}$ molars (WT_E16_1, Homo_E16_1) were aligned to the mm10 mouse genome and visualized using the Integrative Genomics Viewer (IGV) browser (version 2.17.1 01, https://igv.org/). The data range for the coverage track of both samples was adjusted to 0-250. The genomic region (mm10,

chr11:120,653,500-120,662,000), including the *Notum* gene (transcript: NM_175263.4), was captured and annotated.

Mineralization induction and alizarin red S staining

To induce cell differentiation and mineralized nodule formation of the cells, 95% confluent cells were cultured in osteogenic media (OM) containing α-MEM with 5% FBS, 50 μg/ml ascorbic acid, 10 mM β-glycerophosphates, and 10 nM dexamethasone (Sigma Aldrich) for up to 6 days. Mineral nodule formation was observed by staining the cells with 40 mM alizarin red S (pH 4.2) after fixation with 4% PFA for 10 min. The amount of alizarin red S that bound to the minerals was quantified by destaining the samples in 10 mM sodium phosphate containing 10% cetylpyridinium chloride (pH 7.0) for 15 min at room temperature. The amount of alizarin red S in the destaining solution was measured at OD 562 nm.

Analysis of dentin apposition rate

To examine the dentin apposition rate, we performed double fluorescence labeling as described previously (Han et al., 2011). Briefly, calcein (5 mg/kg; Sigma Aldrich) was intraperitoneally administered to mice at P7, followed by second administration of calcein at P14. Mice were sacrificed after 2 days (P16). For statistical analysis, 3 independent littermates were used in the study.

RNA preparation and real-time qPCR (RT-qPCR)

1.0×10^5 cells were seeded on a 35mm culture dish, transfected next day for 24 h and cultured with new media for additional 24 h before RNA preparation. To analyze the effect of soluble Notum, cells were supplemented with recombinant Notum (500 ng/ml; R&D Systems, Minneapolis, MN, USA) for 2 days. Total RNA was prepared using a RNeasy Mini kit (QIAGEN, Valencia, CA, USA) according to the manufacturer's instructions, and cDNA was synthesized from 2 μg of total RNA using Superscript II reverse transcriptase (Invitrogen). Real-time PCR was performed with SYBR Green PCR Master Mix (Applied Biosystems, Warrington, Cheshire, UK) following the manufacturer's protocols. Reaction conditions comprised 40 cycles of 15 sec of denaturation at 95°C and 1 min of amplification at 60°C. All reactions were run in triplicate, and expression was normalized to that of the housekeeping gene *glyceraldehyde-3-phosphate dehydrogenase* (*Gapdh*). Relative levels of transcript expression were quantified using the $\Delta\Delta Ct$ method. The calculation was performed using the *Ct* value of *Gapdh* to normalize the *Ct* value of the target gene in each sample and obtain the ΔCt value, which then was used to compare different samples. Relative mRNA expression was compared in a histogram. Specific primers set used in the analysis are listed in Table S1.

Western blot analysis

Proteins (20 - 50 μg) were dissolved in sample buffer, and electrophoresis was carried out at a current of 25 mA for 2 h. Proteins were transferred from SDS-PAGE onto nitrocellulose membranes (Schleicher & Schuell, Dassel, Germany). Membranes were blocked for 1 h with 5% nonfat dry milk in PBS containing 0.1% Tween-20 (PBS-T) and incubated overnight with antibodies for Notum (Sigma Aldrich), Act. β-Cat (Cell signaling), β-Cat (Thermo Scientific), Osx

(Abcam), Runx2 (Abcam), Bsp (Abcam), Dsp (Santa Cruz Biotechnology), Dmp1 (TaKaRa Bio, Shiga, Japan), collagen I (Abcam), Lox (Proteintech, IL, USA), and β-Actin (Santa Cruz Biotechnology) diluted in PBS-T buffer at 4 °C. After washing, the membranes were incubated with anti-rabbit or mouse-IgG conjugated horseradish peroxidase (Santa Cruz Biotechnology) for 1 h. Labeled protein bands were detected using an enhanced chemiluminescence system (Amersham Biosciences, Buckinghamshire, UK). Protein expression levels were analyzed with the ImageQuanT TL 1D gel analysis program (Amersham Biosciences).

Table S1. Primer sequences for real-time qPCR

	**Sense**	**Antisense**
Col1a1	CCGGAAGAATACGTATCACC	ACCAGGAGGACCAGGAAGTC
Col1a2	CAGCGAAGAACTCATACAGCC	TTGGAGCAGCCATCGACTA
Notum	TACGGAGCTATTGGACGGAC	CGTTCATCTCTTGCCCTGTG
Dkk1	TCAATTCCAACGCGATCAAGA	GGCTGGTAGTTGTCAAGAGTCTGG
Dmp1	AGTGAGTCATCAGAAGAAAGTCAAGC	CTATACTGGCCTCTGTCGTAGCC
Runx2	CCTCTGACTTCTGCCTCTGG	TAAAGGTGGCTGGGTAGTGC
Dspp	AACACATCCAGGAACTGCAGCACA	TGACTCGGAGCCATTCCCATCTCT
Msx2	TTCACCACATCCCAGCTTCT	TTCAGCTTTTCCAGTTCCGC
Bsp	AAAGTGAAGGAAAGCGACGA	GTTCCTTCTGCACCTGCTTC
Gapdh	TGCCCAGAACATCATCCCT	GGTCCTCAGTGTAGCCCAAG

References

Adasooriya, D., Jeong, J.-K., Kyeong, M., Kan, S., Kim, J., Cho, E.-S., & Cho, S.-W. (2024). Notum regulates the cusp and root patterns in mouse molar. *Scientific Reports*, *14*(1), 13633. https://doi.org/10.1038/s41598-024-64340-w

Han, X. L., Liu, M., Voisey, A., Ren, Y. S., Kurimoto, P., Gao, T., Tefera, L., Dechow, P., Ke, H. Z., & Feng, J. Q. (2011). Post-natal Effect of Overexpressed DKK1 on Mandibular Molar Formation. *Journal of Dental Research*, *90*(11), 1312–1317. https://doi.org/10.1177/0022034511421926

Legends for Supporting Information Figures

Figure S1. Comparative analysis of dentin formation in developing mouse teeth. (a) Magnified view of the black dashed boxes in Figure 2a. Asterisks indicate circumpulpal dentin formation resulting from ectopic extracellular matrix deposition by odontoblasts that have lost polarity in *Notum*$^{-/-}$ mice. Initially, this circumpulpal dentin remains separated from the mantle dentin (Md). (b) H&E-stained sagittal tissue sections of molars from *Notum*$^{-/-}$ and *Notum*$^{+/+}$ mice at P14, showing full images of the teeth. Black arrows highlight regions of pronounced dysplastic dentin formation. (c) Micro-CT images comparing molars from *Notum*$^{-/-}$ and *Notum*$^{+/+}$ mice at P35, demonstrating differences in mineralized tissue architecture. (d) H&E-stained sagittal sections of P14 mouse incisors displaying dysplastic dentin formation in *Notum*$^{-/-}$ mice. Each incisor panel represents distinct stages of odontogenesis, from early differentiation to initial secretion and active matrix deposition phases, respectively. Black arrows indicate mantle dentin (Md) in *Notum*$^{-/-}$ mice, while an asterisk (*) highlight ectopic extracellular matrix deposition by

odontoblasts that have lost polarity in *Notum*$^{-/-}$ mice, contributing to dysplastic dentin formation. Double-headed arrows indicate dentin thickness. *Abbreviations*: D, dentin; E, enamel; P, pulp; Ab, ameloblasts; Od, odontoblasts; Md, mantle dentin.

Figure S2. Comparative analysis of gene expression in *Notum*-knockdown odontogenic cells. Quantitative RT-PCR analysis showing relative RNA-expression levels of *Notum* and *Dkk1* in *shNotum* and *shNC* cells. Statistical significance is indicated by *P*-values as shown.

Figure S3. Failure of soluble Notum to restore gene expression in *Notum*-knockdown odontogenic cells. RNA expression levels of each gene were analyzed by quantitative RT-PCR after treating *shNC* and *shNotum* cells with recombinant Notum (500 ng/ml) for 2 days. Statistical significance is indicated by *P*-values as shown.

Figure S4. Original full-size blots of Figure 5a. The following antibodies were used: Notum (SAB3500082, 1:500, Sigma Aldrich), Act. β-Cat (8814, 1:2000, Cell signaling), β-Cat (RB9035, 1:2000, Thermo Scientific), and β-Actin (sc-1616R, 1:2000, Santa Cruz Biotechnology). NS, non-specific.

Figure S5. Original full-size blots of Figure 5b. The following antibodies were used: Osx (ab22552, 1:2000, Abcam), Runx2 (ab23981, 1:1000, Abcam), Bsp (ab52128, 1:1000, Abcam),

Dsp (sc33587, 1:500, Santa Cruz Biotechnology), Dmp1 (M176, 1:500, TaKaRa Bio), and β-Actin (sc-1616R, 1:2000, Santa Cruz Biotechnology).

Figure S6. Original full-size blots of Figure 5e. The following antibodies were used: Collagen I (ab21286, 1:1000, Abcam), Lox (17958-1-AP, 1:1000, Proteintech, IL, USA), and β-Actin (sc-1616R, 1:2000, Santa Cruz Biotechnology).

Figure S1. Comparative analysis of dentin formation in developing mouse teeth

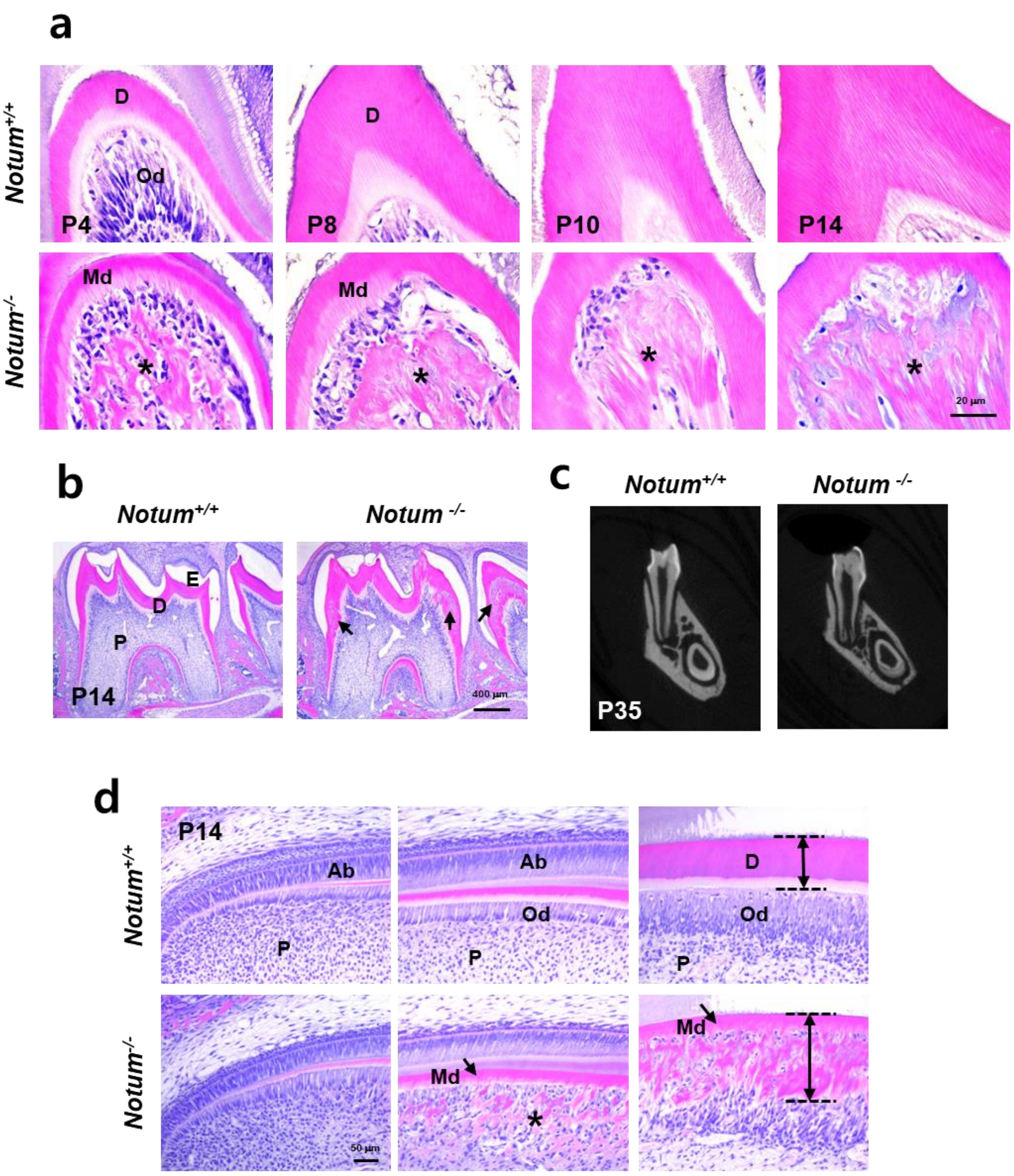

Figure S2. Comparative analysis of gene expression in *Notum*-knockdown odontogenic cells.

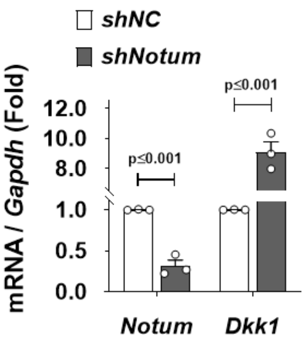

Figure S3. Failure of soluble Notum to restore gene expression in *Notum*-knockdown odontogenic cells.

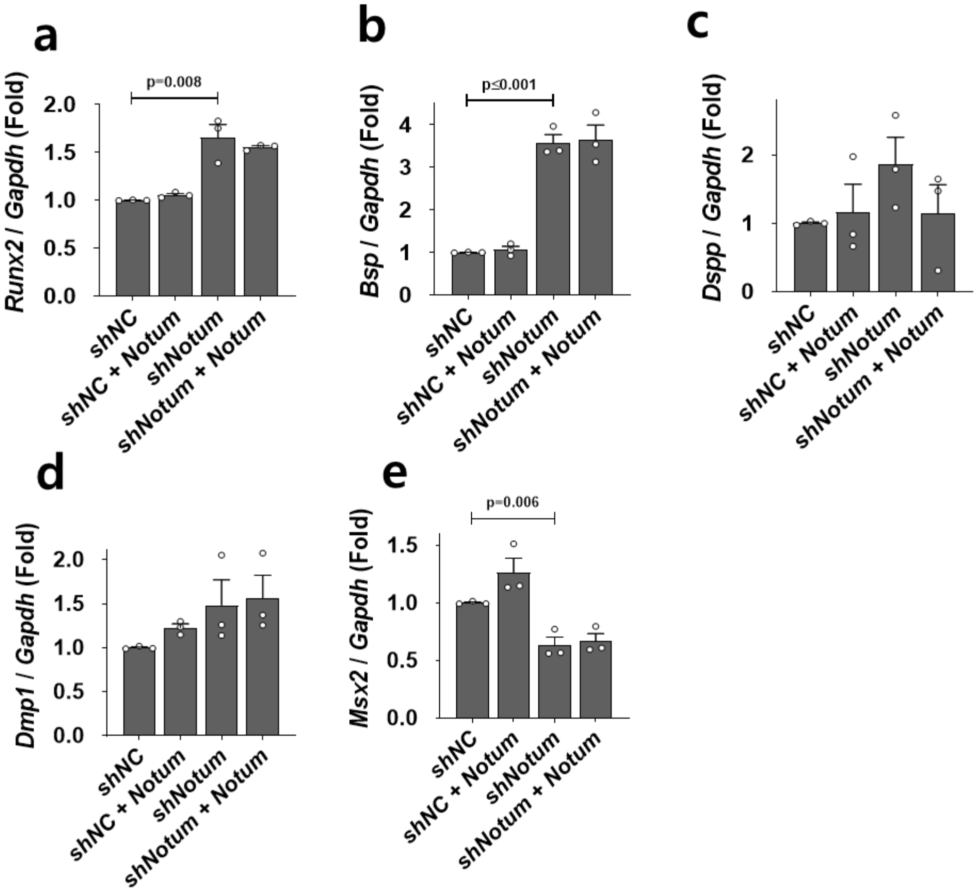

Figure S4. Original full-size blots of Figure 5a.

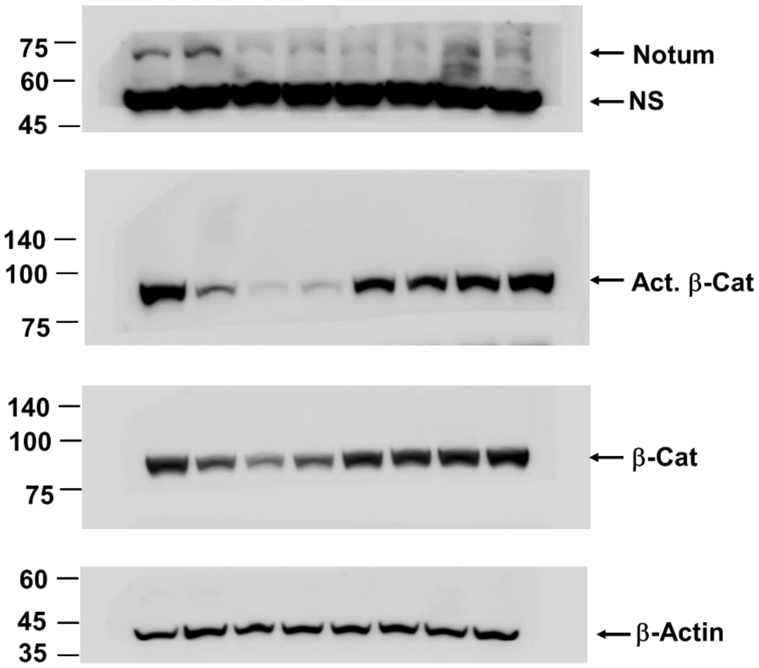

Figure S5. Original full-size blots of Figure 5b.

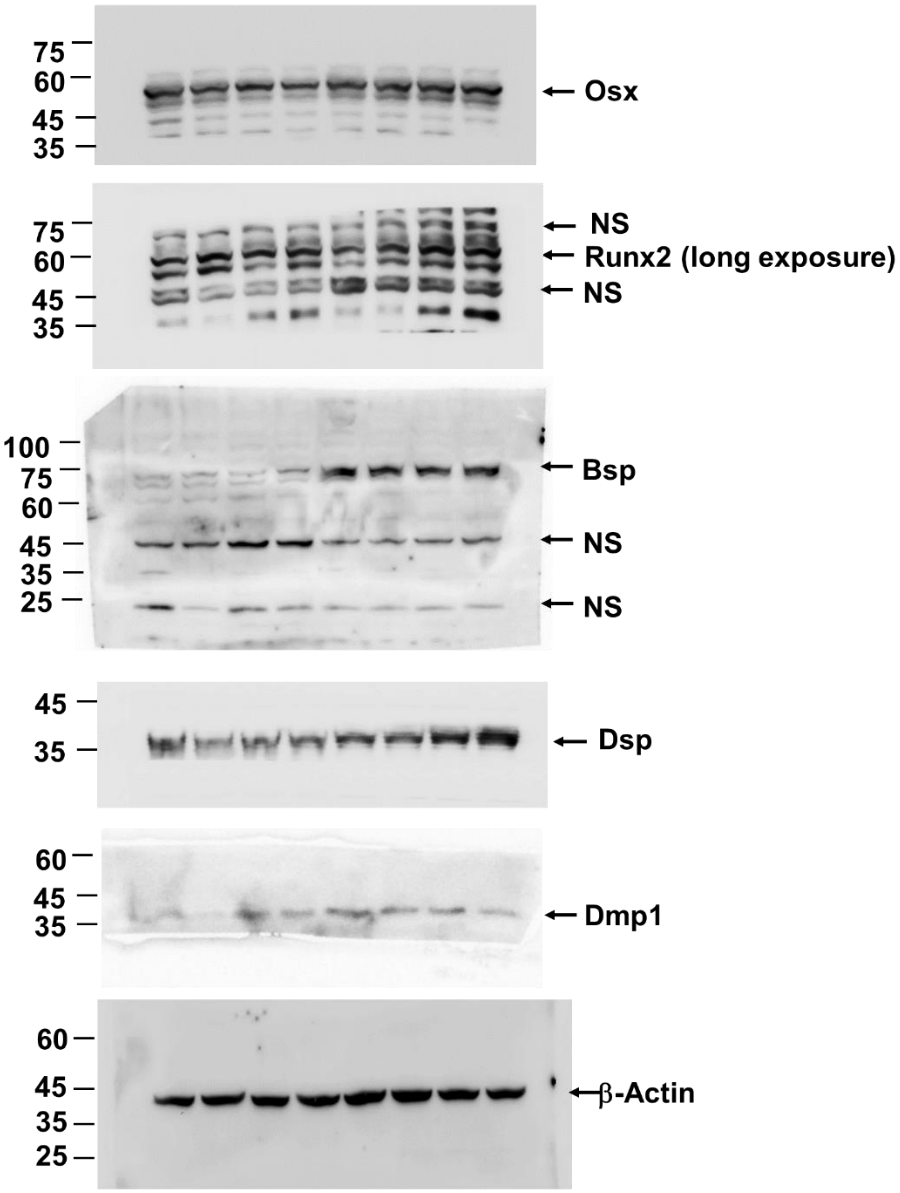

Figure S6. Original full-size blots of Figure 5e.

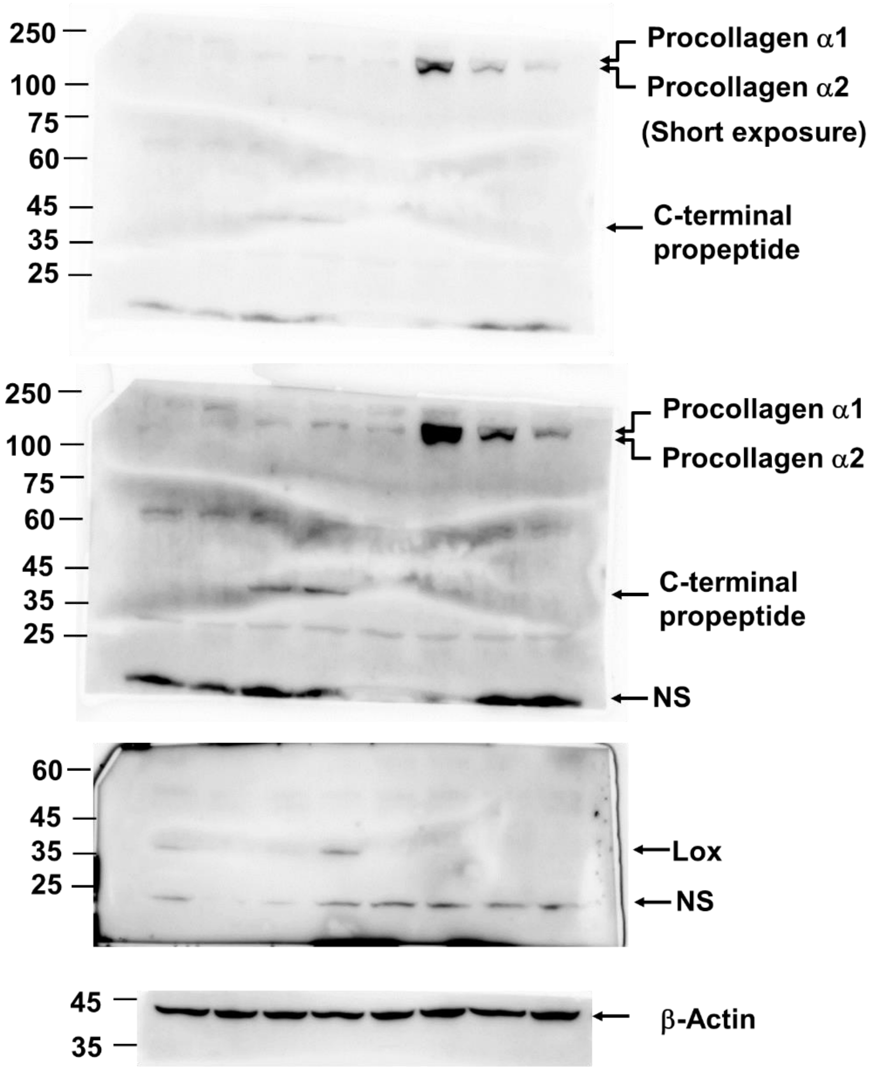

Supplement: Supplementary file 1 — JCP‐24‐2010‐ Supporting Information. [file JCP-240-0-s001.pdf]
